# Molecular characterization of clinical IMP-producing *Klebsiella pneumoniae* isolates from a Chinese Tertiary Hospital

**DOI:** 10.1186/s12941-017-0218-9

**Published:** 2017-06-19

**Authors:** Kaisheng Lai, Yanning Ma, Ling Guo, Jingna An, Liyan Ye, Jiyong Yang

**Affiliations:** Department of Microbiology, Chinese PLA General Hospital, 301 Hospital, 28# Fuxing Road, Beijing, 100853 China

**Keywords:** IMP, *Klebsiella pneumoniae*, Sequence type

## Abstract

**Background:**

IMP-producing *Klebsiella pneumoniae* (IMPKpn) exhibits sporadic prevalence in China. The mechanisms related to the spread of IMPKpn remain unclear.

**Methods:**

Carbapenem non-susceptible *K*. *pneumoniae* isolates were collected from our hospital. The genetic relatedness, antimicrobial susceptibility, as well as sequence types (ST) were analyzed by pulsed-field gel electrophoresis (PFGE), VITEK 2 AST test Kit, and multilocus sequence typing (MLST), respectively. S1-PFGE, Southern blot analysis and multiple PCR amplification were used for plasmid profiling.

**Results:**

Between October 2009 and June 2016, 25 non-repetitive IMPKpn isolates were identified. PFGE results showed that these isolates belonged to 20 genetically unrelated IMPKpn strains. Diverse STs were identified by MLST. Most strains carried *bla*_IMP-4_, followed by *bla*_IMP-1_. Four incompatibility types of *bla*_IMP_-carrying plasmids were identified, which included A/C (n = 2), B/O (n = 2), L/M (n = 1) and N (n = 14), while type of other one plasmid failed to be determined.

**Conclusions:**

The IMPKpn isolates exhibited sporadic prevalence in our hospital. IncN types of plasmids with various sizes have emerged as the main platform mediating the spread of the *bla*_IMP_ genes in our hospital.

## Background

The spread of carbapenemase-producing *Enterobacteriaceae* has been a major challenge both for treatment of individual patients and for policies of infection control [1]. IMP-1 is the first identified metallo-β-lactamase conferring carbapenem resistance and was described in 1988 in a *Pseudomonas aeruginosa* strain in Japan [2]. IMP-type carbapenemases can hydrolyze almost all β-lactams. The popular IMP-type carbapenemases have been widespread in non-fermenting Gram-negative bacilli, including *Pseudomonas aeruginosa*, *Acinetobacter* spp., and members of the *Enterobacteriaceae* family [3]. The *bla*_IMP_ genes are usually located on large plasmids with different replicon types or incompatibility (Inc) types, such as IncA/C, L/M, N, and HI2, which have been commonly associated with the carriage and transmission of various *bla*_IMP_ genes [4–7].

*Klebsiella pneumoniae* is an important pathogen causing various infections. IMP-producing *K*. *pneumoniae* (IMPKpn) isolates have been identified worldwide and caused a few small-scale outbreaks [3, 5, 6, 8, 9]. Up to now, diverse sequence types (ST) of IMPKpn have been reported, including STs 15, 37, 107, 133, 252, 323, 340, 476, 478, 626, 686, 889, 903, 1114 and 1306 [5–11].

In China, IMP-producing *Enterobacteriaceae* isolates have been found across the country [3, 6, 7, 9–21]. Variants of *bla*_IMP_ have been identified in *K*. *pneumoniae*, *Escherichia coli* and *Enterobacter cloacae*. Among IMPKpn, IMP-4 is the most commonly encountered isoform in clinical isolates, followed by IMP-8, IMP-26 and IMP-38 [6, 9–18, 22]. Meanwhile, the IMPKpn isolates exhibit high diversity of sequence type (ST), and IMPKpn STs15, 37, 107, 133, 323, 476, 686, 889, 1114 and 1306 have been recovered [6, 7, 10, 11].

In the present study, a retrospective study of clinical carbapenem-non-susceptible *enterobacteriaceae* isolates from our hospital was performed. In total, 25 IMPKpn isolates were identified in this study. Phenotypic and genotypic characteristics of these isolates were further analyzed.

## Methods

### Bacterial isolates

All clinical *Enterobacteriaceae* isolates were collected from a 4000-bed tertiary-care hospital and were identified by VITEK^®^ MS (bioMérieux SA, Marcy-l’Etoile, France). The isolates exhibiting resistance or intermediate to any one of ertapenem or imipenem or meropenem will be defined as carbapenem non-susceptibility strain, and will be screened for *bla*_IMP_ by PCR and subsequent amplicon sequencing using the primers IMP-1F: 5′-TGAGCAAGTTATCTGTATTC and IMP-1R: 5′-TTAGTTGCTTGGTTTTGATG [11]. *E. coli* ATCC 25922 was used as a quality control strain for antimicrobial susceptibility testing. The *Salmonella* ser. Braenderup strain H9812 was used as a reference standard for pulsed-field gel electrophoresis (PFGE). No ethical approval was obtained for using the clinical samples, because they were collected during routine bacteriologic analyses in public hospitals, and the data were anonymously analyzed.

### Antimicrobial susceptibility testing

**Fig. 1:**
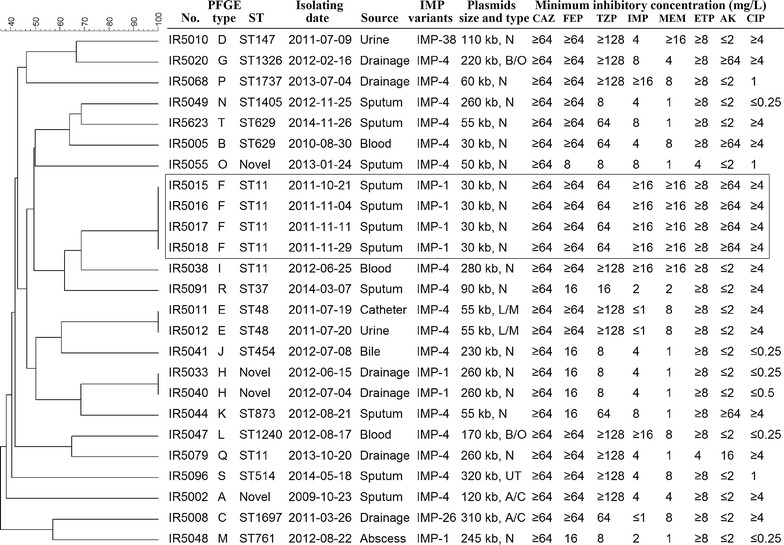
Dendrogram of patterns generated by PFGE of IMP-producing *K. pneumoniae* isolates. The PFGE types, ST, isolating date of the IMPKpn isolates, IMP variants, the size and type of *bla*_IMP_-carrying plasmids and the MICs of common-used antimicrobial agents were list in this figure. The frame indicated the characterization of IMP-producing *K*. *pneumoniae* isolates causing an outbreak. *ST* sequence type, *CAZ* ceftazidime, *FEP* cefepime, *TZP* piperacillin–tazobactam, *IMP* imipenem, *MEM* meropenem, *ETP* ertapenem, *AK* amikacin, *CIP* ciprofloxacin, *UT* unable type

The MICs of clinical commonly used antimicrobial agents (listed in Fig. 1) were measured using VITEK 2 AST-GN09 and AST-GN13 test Kit (bioMérieux, Inc.) according to the manufacturer’s instructions. All susceptibility results were interpreted according to the 2017 CLSI performance standards [23].

### PFGE and MLST analyses

PFGE with *Xba*I was performed for IMPKpn isolates as previously described [24]. MLST was carried out for IMPKpn according to protocols provided on their MLST websites (http://www.pasteur.fr/recherche/genopole/PF8/mlst/Kpneumoniae).

### Plasmid and southern blot analyses

A *bla*_NDM_ probe was generated by labeling the PCR product using the PCR DIG Probe Synthesis Kit (Roche Applied Sciences, Mannheim, Germany). The size and incompatibility types of *bla*_IMP_-carrying plasmids were analyzed by the S1-PFGE, Southern blot and multiple PCR as previously described [24, 25]. The whole genomic DNA including the plasmid of the isolates was digested with 20 U of S1 nuclease at 37 °C for 20 min, and separated by PFGE. Then, the DNA fragments were transferred to positively charged nylon membranes (Roche Applied Science). Hybridization was carried out with the DIG Easy Hyb Granules (Roche Applied Sciences), and the detection was performed using the DIG Nucleic Acid Detection Kit (Roche Applied Sciences).

## Results

### Prevalence and genetic relatedness of IMPKpn

The first IMPKpn isolate in our hospital was identified in October 2009. Since then until June 2016, 25 non-repetitive IMPKpn isolates have been recovered. The source of the IMPKpn isolates included 11 sputum, 6 drainges, 3 bloods, 2 urines, 1 catheter, 1 bile and 1 abscess. The 25 IMPKpn isolates were categorized into 20 PFGE types (type A to T). Type F contained 4 isolates, and types E and H contained 2 isolates, other 16 types contained only 1 isolate (Fig. 1). Isolates with same PFGE type were considered as the same strain. Therefore, a total of 20 IMPKpn strains with no genetic relationship were further analyzed.

### Antimicrobial susceptibility

The antimicrobial susceptibility patterns of the isolates were listed in Fig. 1. All isolates presented resistance to ceftazidime, cefepime and piperacillin–tazobactam, and exhibited heterogeneous resistance patterns to carbapenem, amikacin and ciprofloxacin (Fig. 1).

### *bla*_IMP_ variants

PCR and subsequent amplicon sequence alignment revealed that most strains carried *bla*_IMP-4_, and several *bla*_IMP_ gene sequences displayed 100% identity with the published sequence of the *bla*_IMP-1_ (for 3 strains), *bla*_IMP-26_ (for 1 strains) and *bla*_IMP-38_ (for 1 strains) genes, respectively (Fig. 1).

### MLST

Among 20 IMPKpn strains, a diversity of STs was identified. Three strains were identified as ST11, two strains were defined as ST629, and 12 strains belonged to independent STs. Three novel STs were identified with the allelic profile 18-71-26-125-115-2-51 (type A strain), 42-22-25-96-115-20-49 (type H strain) and 71-1-1-83-16-121-5 (type O strain), respectively (Fig. 1).

### Plasmid analysis

The *bla*_IMP_ genes were located on plasmids with sizes ranged from approximately 30 to 320 kb (Fig. 1). Four incompatibility types of *bla*_IMP_-carrying plasmids including A/C, B/O, L/M and N were identified, whereas type of one plasmid was unable to be determined. In total, 14 IMPKpn strains carried the IncN plasmids with sizes ranging from 30 to 280 kb, while two A/C (120- and 310-kb), two IncB/O (170- and 220-kb) and one IncL/M (55 kb) plasmids were characterized.

## Discussion

The worldwide spread of carbapenemase-producing *K*. *pneumoniae* has been a growing clinical problem and threat to public health [1]. In this study, 25 non-duplicated IMPKpn isolates were collected between October 2009 and June 2016. During this period, 8.5% (537 of 6310) of clinical *K*. *pneumoniae* isolates exhibited carbapenem-resistant phenotypes, and only 0.19% (25/537) of carbapenem-non-susceptible *K. pneumoniae* strains produced IMP. Further analysis showed that the majority of carbapenemase-producing *K*. *pneumoniae* was the KPC-2-producing *K*. *pneumoniae*, followed by OXA-48- and NDM-producing *K*. *pneumoniae* [24–26]. Therefore, IMPKpn exhibited sporadic prevalence in our hospital. In China, IMPKpn isolates have been identified across the country, and the main variants are IMP-4 and IMP-8 [3, 6, 7, 9–21], while IMP-26 and IMP-38 have also been identified [16, 18]. In this study, besides the scattered emergence of IMP-1, IMP-26 and IMP-38, we found that the majority of IMPKpn isolates produced IPM-4 (Fig. 1) which was different from the prevalence of IMP variants that were reported from other Asian regions, where IMP-1 accounted for the majority [3]. Taken together, the source, evolution and dissemination of IMPKpn may be different in different regions.

In this study, PFGE analysis revealed that the majority of IMPKpn isolates belonged to different types, while an outbreak at small scales (type F strain) that covered only four patients was observed (Fig. 1), suggesting that the majority of IMPKpn isolates analyzed in this study were genetically unrelated strains. Meanwhile, most strains exhibited high ST diversity. Only three and two clones of IMPKpn strains belonged to ST11 and ST629, respectively, while other STs were only identified in single strain. Three strains were identified as new clones with novel STs (Fig. 1). Other reports also showed that the STs of IMPKpn are highly dispersed [5–11], suggesting the clonal diversity of IMPKpn isolates. Thus, the IMPKpn exhibited a significant sporadic prevalence across the world. Under the same medical condition and selective pressure, the KPC-2-producing *K. pneumoniae* spread throughout our hospital [24]. However, the IMPKpn strains only exhibited low prevalence and small scale of outbreaks at the same time. A study revealed that some biological characteristics, such as cell motility, secretion, DNA repair and modification, may contribute to the rapid spread of KPC-producing *K. pneumoniae* ST258 and ST11 [27]. Recent genomic analysis of *K. pneumoniae* has established that the genomic background was closely related to the pathogenicity of *K. pneumoniae* [28]. Therefore, the virulence characteristics, rather than their drug-resistant phenotype, may be the major driving force responsible for the emergence and widely spread of antibiotic-resistant pathogens. The urgent efforts are needed to reveal the genetic background of these pathogens, as well as its relationship with their pathogenic and transmission abilities, which is the essential first step to design intervention strategies preventing their spread.

In this study, most of the clinical isolates exhibited low-level resistance or even sensitivity to imipenem and meropenem, but high-level resistance to ertapenem (Fig. 1). Similar results have also been reported in other studies from China [9, 13, 14, 16, 20]. These studies showed that the effects of IMP hydrolase on different carbapenems may be diverse, and therefore, the carbapenem-sensitive phenotype does not mean negative for carbapenemase. In most laboratories in China, the production of carbapenemases was not usually detected by modified Hodge test [23] in clinical IMPKpn isolates with carbapenem-sensitive phenotype. The weak hydrolytic ability of IMPs to carbapenems may lead to an underestimation of the prevalence of IMPKpn isolates. It was reported that some IMP variants (such IMP-26) exhibited increased carbapenem-hydrolyzing activity [29]. However, the IMP-26-producing IMPKpn were sensitive to imipenem in this study (Fig. 1). Another study also showed that the IMP-26-producing IMPKpn displayed only low-level resistance to imipenem [14]. Therefore, the production of IMPs was not the only factor responsible for high-level carbapenem resistance, other resistance mechanisms may also confer this process, which include hyperexpression of efflux systems, and loss of the outer membrane channel OprD that allows the entry of carbapenems into the cell [30]. For clinical IMPKpn isolates, it is less clear to what extent the different resistant mechanisms have impact on the carbapenem-resistant phenotype.

Plasmid analysis in this study showed that the majority (13 of 20) of *bla*_IMP_-carrying plasmids belonged to IncN. Another study in China found the dissemination of IncN *bla*_IMP_-coding plasmids among *K. pneumoniae* of different genotypes [7]. These results suggested that IncN plasmids have a unique advantage and fitness in the spread of *bla*_IMP_ genes. However, because most of the IncN plasmids had different sizes in this study (Fig. 1), it was possible that the dissemination of *bla*_IMP_ genes in our hospital may not mediated by transfer of plasmids but other mechanisms.

The present study has several limitations. First, because this study was retrospective analysis and only limited patient information was available, thus the study focused on the characterization of phenotype and genotype of clinical IMPKpn isolates. Second, the genetic environment of *bla*_IMP_ genes remained to be characterized in our future study. Another limitation of the study included the absence of analyzing other resistance-related determinants, the outer-membrane permeabilities, and efflux systems of IMPKpn isolates. Further studies are needed to address these limitations.
